# Metabolic disturbances potentially attributable to clogging during continuous renal replacement therapy

**DOI:** 10.1186/s40635-023-00581-9

**Published:** 2023-12-21

**Authors:** Mattia M. Müller, Larina Caspar, Onur Sazpinar, Daniel A. Hofmaenner, Rolf Erlebach, Rea Andermatt, Christoph C. Ganter, Reto A. Schuepbach, Pedro D. Wendel-Garcia, Sascha David

**Affiliations:** 1 Institute of Intensive Care Medicine, University Hospital Zurich, Rämistrasse 100, 8091 Zurich, Switzerland; 2 Interdepartmental Division of Critical Care Medicine, University of Toronto, Toronto, Canada; 3 Department of Nephrology, Hannover Medical School, Hanover, Germany

**Keywords:** Dialysis, CRRT, Hypertriglyceridemia, ICU, COVID-19

## Abstract

**Background:**

Clogging is characterized by a progressive impairment of transmembrane patency in renal replacement devices and occurs due to obstruction of pores by unknown molecules. If citrate-based anti-coagulation is used, clogging can manifest as a metabolic alkalosis accompanied by hypernatremia and hypercalcemia, primarily a consequence of Na_3_Citrate infusion. An increased incidence of clogging has been observed during the COVID-19 pandemic. However, precise factors contributing to the formation remain uncertain. This investigation aimed to analyze its incidence and assessed time-varying trajectories of associated factors in critically ill patients on continuous renal replacement therapy (CRRT).

**Methods:**

In this retrospective, single-center data analysis, we evaluated COVID-19 patients undergoing CRRT and admitted to critical care between March 2020 and December 2021. We assessed the proportional incidence of clogging surrogates in the overall population and subgroups based on the specific CRRT devices employed at our institution, including multiFiltrate (Fresenius Medical Care) and Prismaflex System (Baxter). Moderate and severe clogging were defined as Na > 145 or ≥ 150 mmol/l and HCO_3_^−^ > 28.0 or ≥ 30 mmol/l, respectively, with a total albumin-corrected calcium > 2.54 mmol/l. A mixed effect model was introduced to investigate factors associated with development of clogging.

**Results:**

Fifty-three patients with 240 CRRT runs were analyzed. Moderate and severe clogging occurred in 15% (8/53) and 19% (10/53) of patients, respectively. Twenty-seven percent (37/136) of CRRTs conducted with a multiFiltrate device met the criteria for clogging, whereas no clogging could be observed in patients dialyzed with the Prismaflex System. Occurrence of clogging was associated with elevated triglyceride plasma levels at filter start (*p* = 0.013), amount of enteral nutrition (*p* = 0.002) and an increasing white blood cell count over time (*p* = 0.002).

**Conclusions:**

Clogging seems to be a frequently observed phenomenon in critically ill COVID-19 patients. The presence of hypertriglyceridemia, combined with systemic inflammation, may facilitate the development of an impermeable secondary membrane within filters, thereby contributing to compromised membrane patency.

**Supplementary Information:**

The online version contains supplementary material available at 10.1186/s40635-023-00581-9.

## Background

**Fig. 1:**
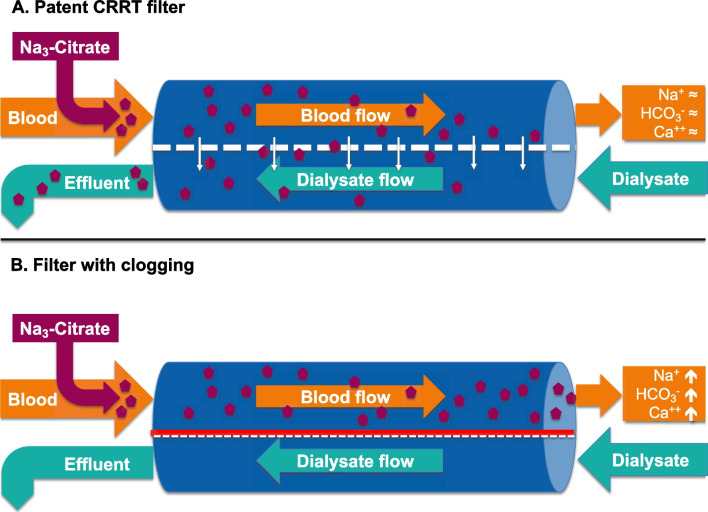
Mechanism of clogging. Representation of a patent hemodialysis filter **A** with clearance up to 60% of calcium–citrate complexes. **B** Occurrence of clogging leads throughout formation of an impermeable secondary membrane to an impairment of clearance capacity of the filter with consecutive rise of sodium, bicarbonate and calcium in the patient

Acute kidney injury (AKI) is a common complication in patients with severe COVID-19, affecting up to 50% of critically ill individuals[1]. Of those, up to 68.3% are submitted to continuous renal replacement therapy (CRRT) [2]. Regional citrate anticoagulation (RCA) and regional or systemic adjunction of unfractioned heparin are among the most frequently used strategies for an efficient inhibition of the plasmatic coagulation cascade to prevent clot formation. Current guidelines recommend RCA as the primary anti-coagulatory agent for the prevention of filter clotting [3–5]. Besides the well-known circuit clotting, the occurrence of the so-called *clogging* is a still under-recognized phenomenon, leading to a subtle loss of clearance capacity. Clogging refers to an isolated reduction of filter transmembrane patency, mediated through molecules covering the filter surface and thereby obstructing the pores, but not the capillary per se. Hyperlipidemia, activated coagulation, systemic inflammation and elevated blood viscosity secondary to increased levels of proteins have all been suspected as potential contributing factors [6–11]. First of all, clogging leads to a progressive loss of dialysis efficacy. When an RCA-based CRRT is used it also leads to accumulation of citrate in the patient with consecutive electrolyte and acid–base disturbances. Usually, citrate is infused as trisodium citrate (Na_3_Citrate) in the hemofilter circuit before the dialyzer and leads to the formation of calcium–citrate complexes. Sixty percent of these are eliminated directly through the dialysis membrane. Residual components enter the systemic circulation and are metabolized to bicarbonate within the Krebs cycle of hepatic, muscular and renal tissue [12]. Manifestation of clogging along the filter reduces the amount of removed calcium–citrate complexes with a consecutive higher load within the systemic circulation, resulting in a typical triad of metabolic alkalosis with hypercalcemia and hypernatremia [13]. Figure 1 provides a schematic representation of clogging.

During the COVID-19 pandemic, clogging gained the awareness of health care practitioners due to presumably an increased incidence [11, 13]. So far, the pathological mechanism and attributed factors contributing to the formation of clogging have not been evaluated sufficiently. The aim of this study was to assess the incidence of clogging and detect associated factors for the development of preventive strategies in the future.

## Methods

### Patients

The study was conducted in accordance with the Declaration of Helsinki and reviewed by the ethical commission board of the canton of Zurich (BASEC-Nr. 2022-00285). The COVID-19 cohort database of the Institute of Intensive Care Medicine of the University Hospital of Zurich (USZ) was reviewed for patients hospitalized at the ICU between March 2020 and December 2021 with a positive SARS-CoV-2 polymerase chain reaction (PCR) test on naso-pharyngeal swab, trachea–bronchial secretion or broncho-alveolar lavage fluid sample, and requirement of CRRT. Patients were excluded if age was younger than 18 years, in the presence of an oral or written refusal of further use of medical-related data for research purposes, if CRRT was discontinued before ICU admission, or if patients received solely sustained low efficiency dialysis (SLED) or intermittent hemodialysis (IHD). Individuals with metabolic derangements conforming to the definition of clogging already at the beginning of the CRRT run (first blood gas analysis upon initiation of CRRT) were not included in the further assessment, since no distinction between pre-existing derangements without new onset of clogging and recurrent clogging could be made. The exact definition of clogging is presented in the dedicated section below. Individuals only receiving heparin-based regime for prevention of filter clotting were not considered for analysis, since clogging-induced metabolic disturbances only occur with citrate anti-coagulation.

### Data collection

Electronical charts were reviewed for fully documented CRRT runs, defined as a complete electronically registered filter’s life span cycle, which lasts up to maximal 72 h, according to the institutional protocol. CRRT runs with missing starting or ending times or without transmission of device’s setting into the electronical patient data management system were excluded. Patient characteristics such as age, gender, comorbidities as well as type of hemofilter device, hemofilter settings and laboratory parameters were extracted from the electronical data system for further analysis.

### Definition of clogging for the purpose of this study

Laboratory parameters were assessed for new onset of hypernatremia and metabolic alkalosis in two consecutively measured blood gas analysis, alongside elevated levels of total albumin-corrected calcium (corrected_Alb_ Ca) during CRRT. Moderate clogging was arbitrarily defined as sodium levels > 145 mmol/l, HCO_3_^−^ > 28.0 mmol/l and a total corrected_Alb_ Ca > 2.54 mmol/l. Severe clogging was considered an increase of sodium ≥ 150 mmol/l, HCO3^-^ ≥ 30.0 mmol/l and corrected_Alb_ Ca > 2.54 mmol/l. Filter runs which meet only temporarily the criteria for clogging but are normalized until the end of the filter run is reached were categorized as transient clogging and were handled in the primary analysis likewise filters which showed clogging until the end of the run.

### Data analysis

Patients were stratified according to the predefined criteria into a non-clogging and a clogging group. The latter was subdivided into moderate clogging and severe clogging. Shapiro–Wilk test was used to assess for normal distribution and student’s *t* test or Mann–Whitney–Wilcoxon test were applied to identify difference in patient characteristics between individuals with and without clogging. The incidence of clogging was compared between the two standard CRRT devices utilized at our institution, namely, multiFiltrate (mFT, Fresenius Medical Care, Bad Homburg, Germany) and Prismaflex System (PFS, Baxter, Deerfield, USA), employing Fisher's exact test. Continuous measured filter settings (blood flow, dialysate flow, substitute flow, ultrafiltration) and laboratory parameters (hematological, chemical and coagulation) were summarized in daily representative figures, selecting the first value measured after every 24 h (timepoint 0, 24 and 48 h). Measurements of blood gas analysis and substitution rate of calcium were summarized in 3 values per day selecting the first registered parameter after every 8 h. Total amount of propofol from ICU admission until start of CRRT was assessed. The accumulative amount of applied propofol and enteral nutrition in the last 24 h before filter start (timepoint T0) and for every 24 h of filter running time (timepoint 24 and 48 h) were calculated additionally.

Parameters reflecting clogging were compared at filter start (T0) with student’s *t* test or Mann–Whitney–Wilcoxon test between clogging and non-clogging filters to assess baseline levels. A subgroup analysis was performed analyzing the first CRRT run of each patient manifesting clogging compared to all non-clogging filter separately (first-clogging subgroup), to assess characteristics associated with the very first formation of clogging. To address potential confounders arising from prior CRRTs, we evaluated an additional subgroup comprised of the first CRRT run of each patient (first-CRRT subgroup).

The temporal relationship of different variables hypothetically associated with clogging was studied by means of linear mixed-effect models, considering clogging/non-clogging and time as independent variables with interaction effect. For variables with a temporal resolution higher than 24 h, time was modelled by means of natural cubic splines. As random effects per patient random intercepts and intra-patient random slopes were entered into the model. Effects of clogging vs. non-clogging on the specified variables are presented as mean differences with 95% confidence interval [95%CI] of intercepts (ΔI) and regression coefficients (Δβ). Due to the low sample size, the regression model was not applied for the first-filter subgroup.

To reflect the competing structure of clogging events, CRRT stopping and a patient’s death, cumulative incidence curves were constructed to explain and describe the temporal relationship of clogging events.

Statistical analysis was performed through a fully scripted data management pathway using the R environment for statistical computing version 4.2.0. A two-sided *p* < 0.05 was considered statistically significant. Values were expressed as median and interquartile range [IQR] or minimal (min.) and maximal (max.) values where appropriate.

## Results

### Patient characteristics

**Fig. 2:**
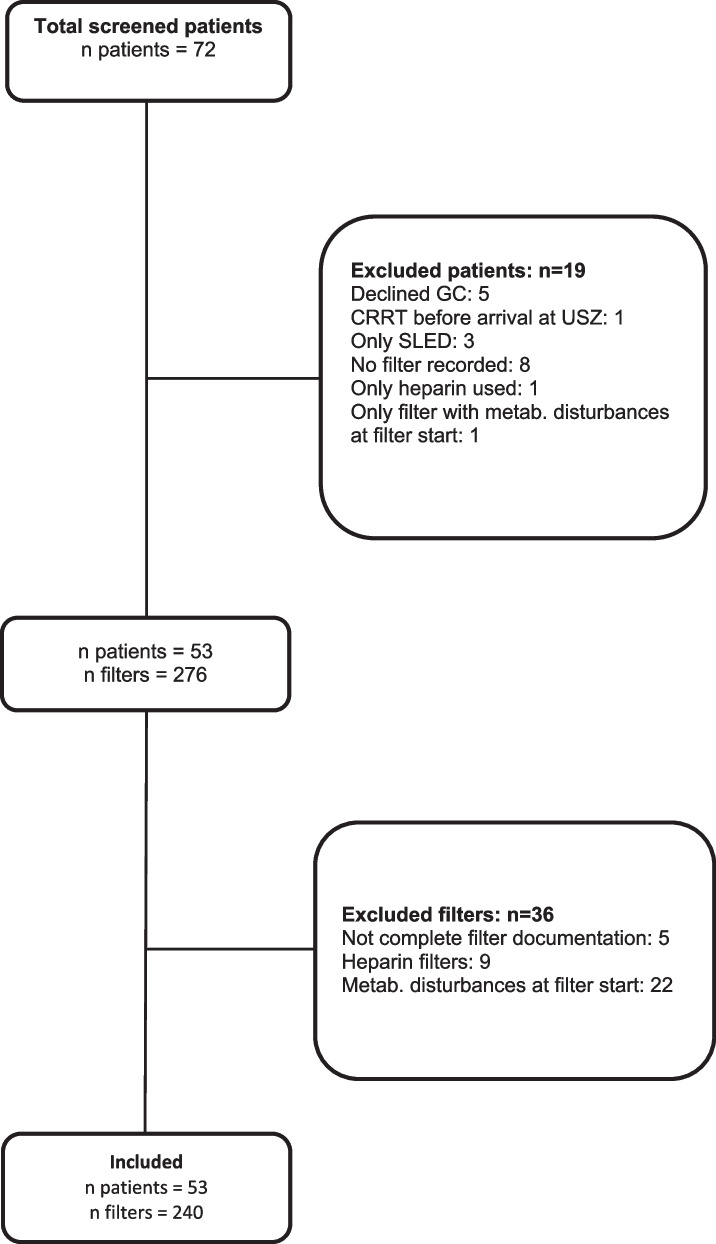
Screening procedure. GC = general consent for the use of health-related date for research purposes

Seventy-two patients were treated between March 2020 and December 2021 with a diagnosis of COVID-19 and concurrent requirement of CRRT. After separation of individuals not meeting criteria for eligibility, 53 patients with a total of 240 CRRT runs were included in the final analysis. A detailed presentation of excluded patients and CRRT runs is provided in Fig. 2, characteristics of included patients are summarized in Table 1 and Additional file 1: Table S1.

**Table 1:** Patient characteristics

	Overall	Non-clogging	Clogging	*p* value
	*n* = 53	*n* = 35	*n* = 18	(Non-clogging vs. clogging)
Demographics				
Age	61.0 [56.0–69.0]	61.0 [56.0–70.0]	63.0 [58.2–67.5]	0.851
Weight	89.0 [75.0–100.0]	85.0 [73.9–100.0]	90.0 [80.0–109.0]	0.387
Height	175.0 [165.0–181.0]	173.0 [160.0–180.0]	177.5 [170.0–184.8]	**0.049**
Male	39 (74)	24 (69)	15 (83)	0.409
SOFA Score	14.0 [12.0–16.0]	13.0 [10.5–15.0]	15.0 [14.2–17.8]	**0.006**
SAPS II	55.0 [40.0–65.0]	50.0 [37.0–63.5]	60.5 [52.0–67.2]	0.126
APACHE II	23.0 [19.0–29.0]	22.0 [16.0–28.0]	26.0 [23.0–28.8]	0.098
Comorbidities				
Ischemic Heart Disease	8 (15)	7 (20)	1 (6)	0.324
Chronic Heart Failure	7 (13)	2 (6)	5 (28)	0.069
Peripheral Vascular Disease	3 (6)	2 (6)	1 (6)	1
Chronic Arterial Hypertension	17 (32)	12 (34)	5 (28)	0.865
Diabetes mellitus Type 1 or 2	14 (26)	10 (29)	4 (22)	0.867
Diabetes mellitus with End Organ Damage	1 (2)	1 (3)	0 (0)	1
Cerebrovascular Disease	5 (9)	3 (9)	2 (11)	1
Chronic Obstructive Pulmonary Disease	3 (6)	3 (9)	0 (0)	0.515
Peptic Ulcer Disease	1 (2)	1 (3)	0 (0)	1
Mild Liver Disease	2 (4)	2 (6)	0 (0)	0.785
Moderate to Severe Liver Disease	3 (6)	2 (6)	1 (6)	1
Moderate to Severe Chronic Kidney Disease	8 (15)	6 (17)	2 (11)	0.86
Solid Tumor (Localized)	5 (9)	4 (11)	1 (6)	0.844
Solid Tumor (Metastatic)	1 (2)	1 (3)	0 (0)	1
Leukemia	3 (6)	3 (9)	0 (0)	0.515
Lymphoma	1 (2)	1 (3)	0 (0)	1
Immunisupression	4 (8)	3 (9)	1 (6)	1
Organ support and treatment during ICU stay				
Organ support				
Vasopressors	53 (100)	35 (100)	18 (100)	1
HFO/NIV	18 (34)	13 (37)	5 (28)	0.707
IMV	53 (100)	35 (100)	18 (100)	1
ECCO2R	3 (6)	2 (6)	1 (6)	1
ECMO	13 (25)	7 (20)	6 (33)	0.465
Prone Position	41 (77)	26 (74)	15 (83)	0.69
iNO	19 (36)	11 (31)	8 (44)	0.527
Drug Therapies				
Corticosteroids	37 (70)	26 (74)	11 (61)	0.501
Tocilizumab	2 (4)	2 (6)	0 (0)	0.785
Hydroxychloroquine	11 (21)	7 (20)	4 (22)	1
Ritonavir/lopinavir	4 (8)	3 (9)	1 (6)	1
Remdesivir	22 (42)	18 (51)	4 (22)	0.08
Cytosorb	1 (2)	0 (0)	1 (6)	0.732
PEX	1 (2)	1 (3)	0 (0)	1
Baricitinib	1 (2)	1 (3)	0 (0)	1
Outcome parameters				
LOS ICU	24.0 [12.0, 32.0]	23.0 [12.0, 32.0]	29.5 [13.5, 45.2]	0.198
LOS Hospital	41.0 [28.5, 44.2]	36.0 [26.0, 42.0]	42.0 [40.0, 45.0]	0.53
Survivor 28 days	20 (40)	12 (36)	8 (47)	0.67
Survivor 6 months	10 (23)	6 (21)	4 (27)	0.945

### Dialysis filters

The 240 CRRT runs analyzed had a median filter lifespan of 58.3 h (min. 0.2 h, max 78.9 h). Median number of filters per patient was 4 (min. 1, max. 21). In 32 cases, a mFT device with AV1000 filters (Fresenius Medical Care) and continuous veno-venous hemodialysis (CVVHD) modality was used; 18 patients were treated with continuous veno-venous hemodiafiltration (CVVHDF) using PFS with an AN69 Dialyzer membrane (Baxter), in 3 individuals both devices were applied. The choice between treatment with mFT or PFS was contingent upon the specific ICU to which patients were admitted within the Institute and the availability of relevant equipment at that local facility.

Trisodium–citrate was used for RCA in all included CRRT runs. Institutional dialysis protocols are provided in the Additional file 1: Tables S2 and S3**.**

**Fig. 3:**
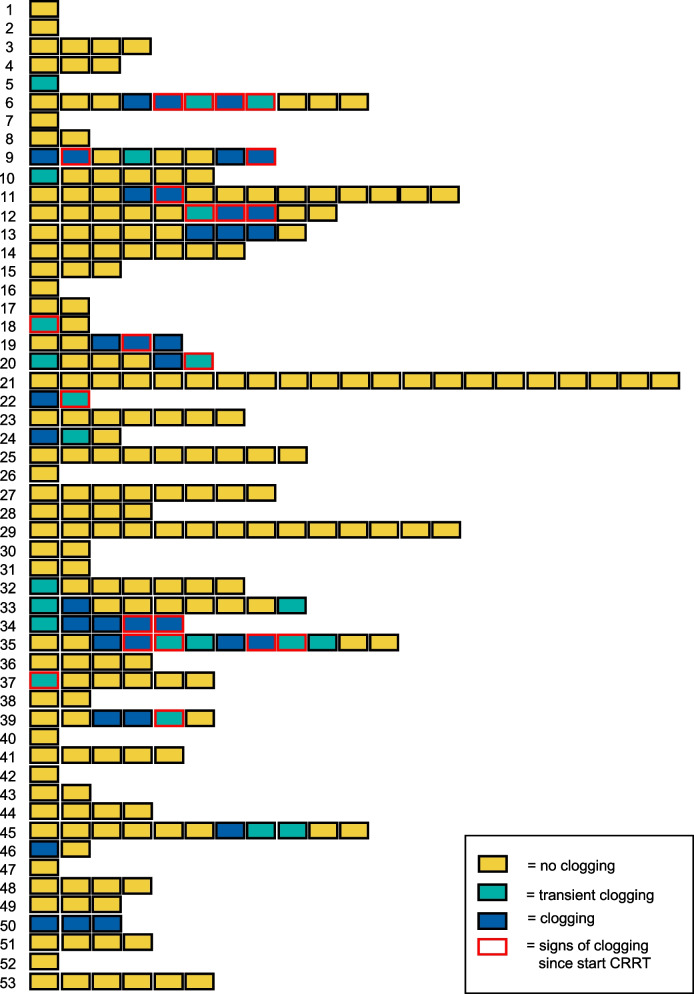
Patients and individually conducted CRRT runs. Numeration on the left side represent patients, each square is a conducted CRRT run without clogging (yellow), transient metabolic disturbances indicative for clogging (turquoise) and persistent signs of clogging until end of filtration (blue). CRRTs with metabolic disturbances already in the first measurements were not included in the final analysis, since no distinguishment between new rapid onset of clogging and pre-existing metabolic alterations without new clogging could be made (red boarders)

There was no significant difference in filter lifespan between mFT and PFS devices (51 h [26.7–67.5] vs. 65 h [40.9–67.8], *p* = 0.085). Figure 3 presents an overview of patients with individually conducted CRRT runs and the occurrence of clogging.

### Incidence of clogging

Thirty-four percent (18/53) of all patients subjected to CRRT showed at least one episode of metabolic derangements consistent with clogging, in 15% (8/53) a moderate and in 19% (10/53) a severe manifestation was present. In 11 patients, metabolic disturbances were observed during the initial CRRT run, while in 7 cases, clogging occurred in a subsequent run. Analyzing the 240 individually conducted CRRT runs demonstrated a 15% (37/240) incidence of clogging, 10% (25/240) moderate and 5% (12/240) severe. Clogging occurred on average 20.1 h [9.4–33.8] after filter start.

**Fig. 4:**
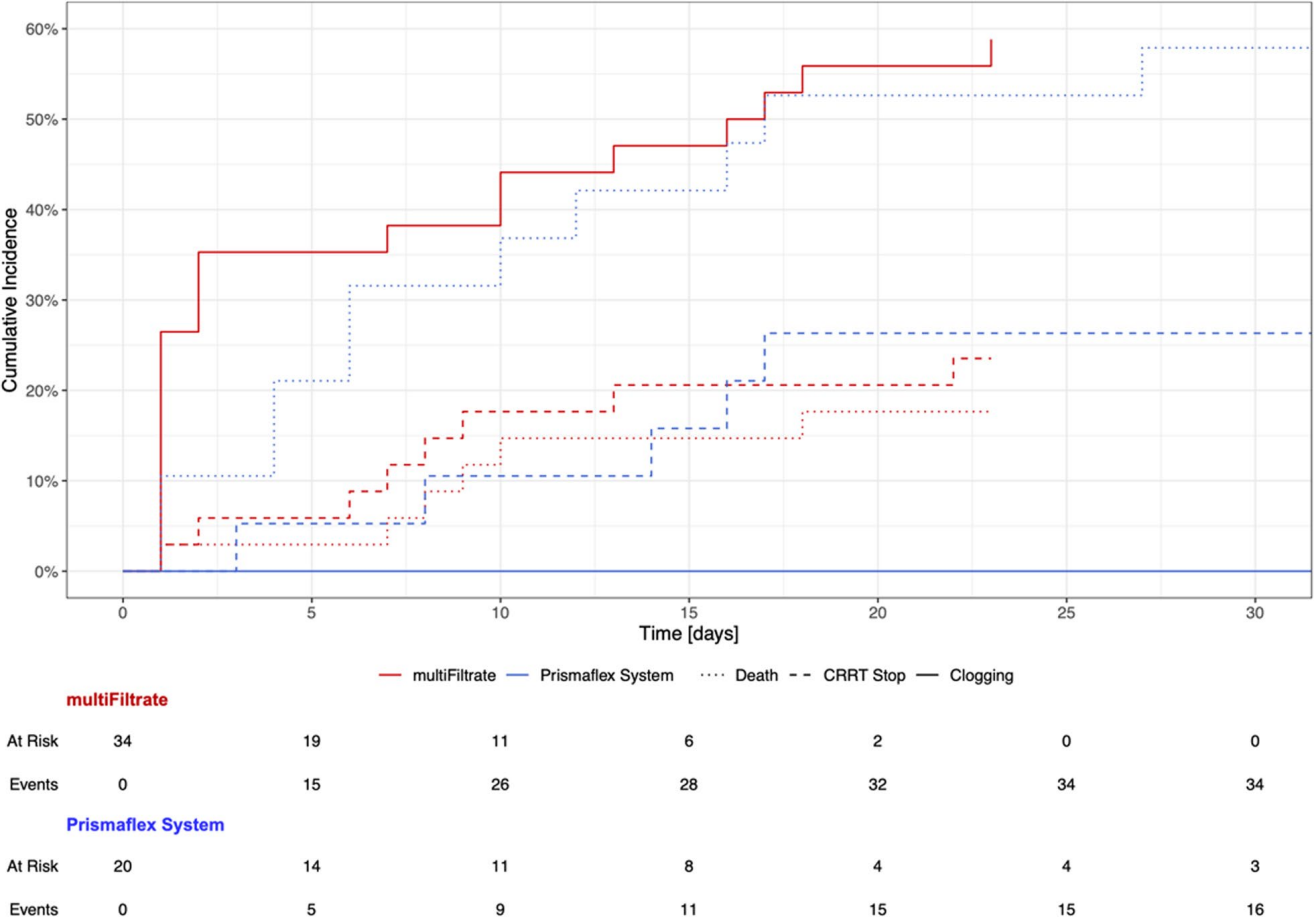
Time-event analysis for clogging of two studied CRRT devices. Patients submitted to CRRT with an mFT device and CVVHD modality had an increased risk to develop clogging during duration of treatment (solid red line). No signs of clogging could be observed in patients treated with CVVHDF using PFS devices (solid blue line). Competing events (discontinuation of CRRT and death) are represented as dashed and dotted lines

Whereas clogging affected 51% (18/35) of patients and 27% (37/136) of all individual CRRT runs conducted with mFT devices, none of the patients treated with the PFS device showed metabolic derangements indicative of clogging (0/21 patients, 0/104 filters; mFT vs. PFS *p* < 0.001, Fig. 4).

### Longitudinal course of metabolic derangements

**Fig. 5:**
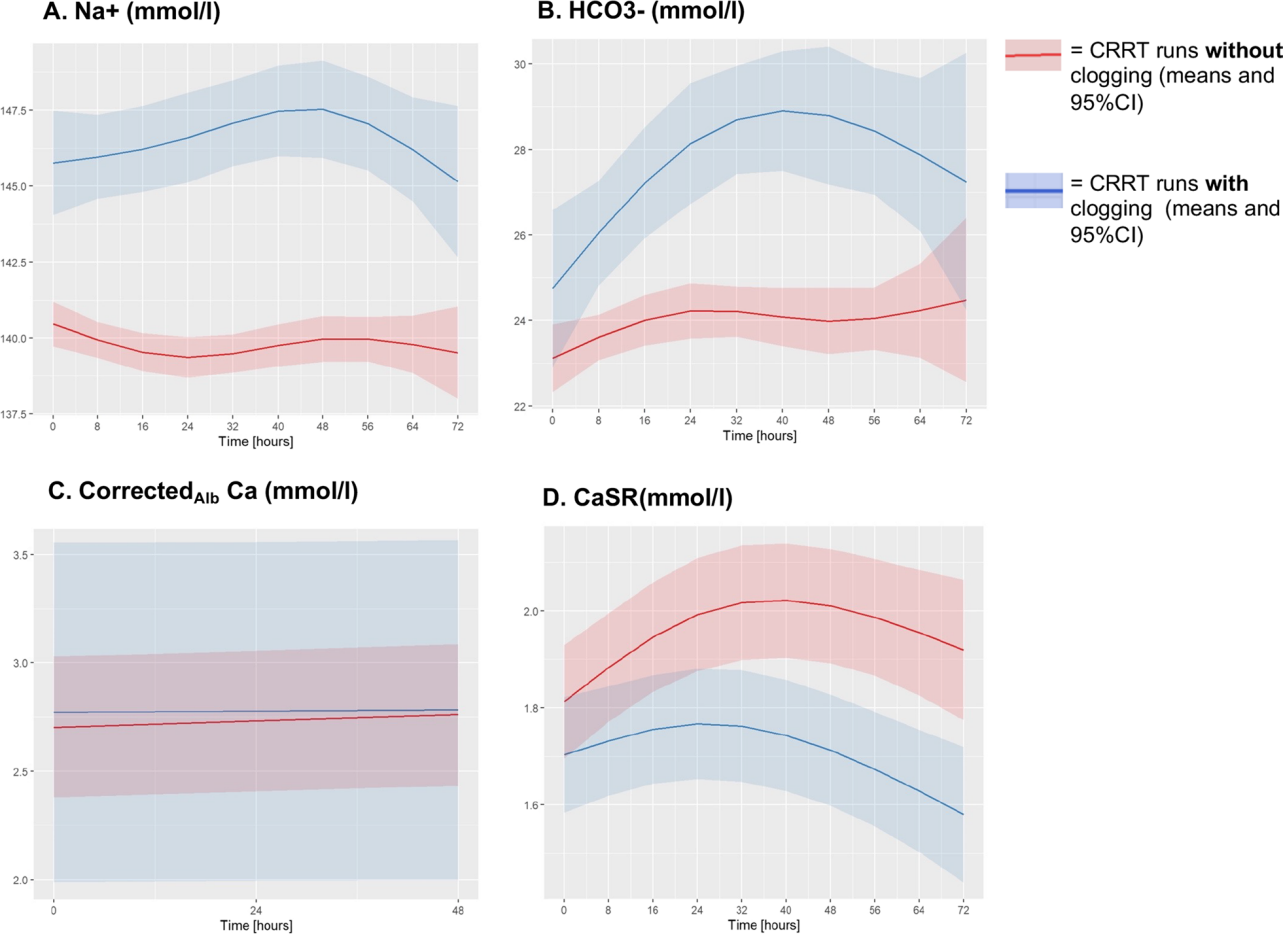
Time-varying trajectories of metabolic alterations. The figure summarizes the course of metabolic markers over time for every conducted filter run. Only values were included, which were measured during a CRRT run. Maximal run time was 72 h as per institutional protocol. Clogging filters (blue line) showed persistently higher sodium concentrations in the plasma **A** with increasing bicarbonate levels over time **B**. Corrected_Alb_ Ca was only slightly increased in filters allocated to the clogging group **C**. Whereas calcium substitution (CaSR) did not differ between overall clogging and non-clogging filters at the beginning of CRRT, a decline of substitution rate could be observed in filters with first onset of clogging (first-clogging, blue line in **D**)

As per definition, filters with clogging were associated with higher levels of plasma sodium compared to non-clogging filters. This distinction was already present at filter start (T0: *p* < 0.001) in the overall population but could not be observed in the subgroup of first-filters (T0: *p* = 0.236). Correspondingly, the mixed-effect model confirmed this significant effect of clogging on the intercept (ΔI: *p* < 0.001) without any impact on time-dependent trajectories (Δβ_1_: *p* = 0.117). Parameters of metabolic disturbances at first measurement after initiation of filtration (T0) are presented in Table 2, time-varying trajectories are visualized in Fig. 5, results of the mixes-effect model are represented in the Additional file 1: Table S4.

**Table 2:** Parameters of metabolic disturbances at start of CRRT runs

	Markers of clogging at the first measurement after start CRRT (T0)				Subgroup first-clogging (T0)				Subgroup first-CRRT run(T0)			
	Overall	Non-clogging	Clogging	*p* value (Non-clogging vs. clogging)	Overall	Non-clogging	Clogging	*p* value (Non-clogging vs. clogging)	Overall	Non-clogging	Clogging	*p* value (Non-clogging vs. clogging)
Sodium (mmol/l)	141	140	145	** < 0.001**	140	140	146	** < 0.001**	146	145	146	0.236
	[137–145]	[137–144]	[144–147]		[137–144]	[137–144]	[144–148]		[143–149]	[142–148]	[146–150]	
Bicarbonate (mmol/l)	23	23	25	** < 0.001**	23	23	24	**0.009**	21	21	24	0.138
	[21–24]	[21–25]	[24–27]		[21–25]	[21–25]	[23–27]		[20–24]	[20–23]	[20–26]	
CorrectedAlb Ca (mmol/l)	2.54	2.53	2.55	0.383	2.54	2.43	2.56	0.548	2.54	2.45	2.5	0.2
	[2.44–2.65]	[2.42–2.64]	[2.46–2.66]		[2.42–2.64]	[2.42–2.64]	[2.42–2.66]		[2.34–2.56]	[2.32–2.55]	[2.41–2.61]	
CaSR (mmol/l)	1.7	1.7	1.7	0.792	1.7	1.7	1.7	0.406	1.7	1.7	1.7	0.602
	[1.7–1.7]	[1.7–1.7]	[1.7–1.7]		[1.7–1.7]	[1.7–1.7]	[1.7–1.7]		[1.7–1.7]	[1.7–1.7]	[1.7–1.7]	

Table 2 illustrates median levels of markers employed for clogging detection at the baseline, which corresponds to the first measurement taken after initiating the CRRT run, for the entire study population. In addition, two distinct subgroup analyses were performed

In the first subgroup analysis, CRRT runs, where clogging first became evident for each individual patient were compared to all runs without clogging (referred to as the "first-clogging subgroup")

The second subgroup consisted of the initial CRRT run for each patient, known as the "first-CRRT subgroup." Notably, differences in marker levels observed at the onset of filter usage within the overall study population were not evident in the first-CRRT subgroup

Median bicarbonate levels in the plasma at beginning of CRRT runs were higher in filters with clogging compared to non-clogging filters (*p* < 0.001). This difference could not be observed in the subgroup of first-filters (*p* = 0.138) and was not reflected in the mixed-effect model (ΔI: *p* = 0.111). Time-dependent trajectories of bicarbonate levels showed an increase over time in association with clogging, with peak levels 40 h after filter start.

Corrected_Alb_ Ca levels were on average numerically higher in the clogging group but failed overall statistical significance compared to non-clogging filters.

Regarding ionized calcium levels in the plasma, no difference could be detected between groups. However, since systemic ionized calcium levels are continuously corrected during CRRT in a nurse-driven approach, calcium substitution rate (CaSR) was analyzed additionally. CaSR was comparable in clogging and non-clogging filters at start (ΔI: *p* = 0.784). In both groups CaSR increased initially but reached on average the highest rate in clogging filters after 24 h, followed by a drop, whereas substitution rate of non-clogging CRRTs peaked after 40 h. The peak value at 24 h in clogging filters corresponds approximately to the timepoint of average occurrence of clogging. Absolute substitution rate at time of clogging was 1.9 mmol/l [1.7–2.1].

### Transmembrane pressure (TMP) and association with metabolic derangements

Metabolic disturbances, which were used as criteria to define clogging, were associated with an increase in TMP (ΔI: *p* < 0.004). However, the interaction between time and clogging revealed a reduction in TMP over time in filters classified as clogging (Δβ1: *p* = 0.01; Δβ2: *p* < 0.001, Δβ3: *p* = 0.001). The use of PFS was notably linked to a significant increase in TMP levels (*p* < 0.001). Additional information depicting the time-dependent TMP trajectories for both non-clogging and clogging filters, as well as for the two devices, can be found in the Additional file 1: Fig. S1.

### Association of metabolic alterations with accumulative citrate exposure and CRRT dose

Clogging (*p* < 0.001) as well as type of device (*p* < 0.001) were both associated with increased citrate exposure, with higher citrate targets during filtration with mFT devices compared to the PFS. CRRT dose did not differ between filters attributed to clogging compared to non-clogging filters (Δβ: *p* = 0.186). Regression models are presented within the supplement section in the Additional file 1: Table S5.

### Impact of clogging on dialysis efficacy

Creatinine decreased linearly during CRRT without any differences between groups (ΔI: *p* = 0.72, Δβ: *p* = 0.257). Clearance of urea was numerically lower in clogging compared to non-clogging filters but failed statistical significance. The phenomenon was more evident assessing filters of the first-clogging group which showed a significant reduction in the clearance of plasma urea over time in association with clogging (Δβ: *p* = 0.014). Additional file 1: Table S6 summarizes results of the mixed-effect model of the trajectories of renal biomarkers and factors with a potential to provoke clogging.

### Potential clogging provoking factors

#### Inflammation

White blood cell counts (WBCs), C-reactive protein (CRP), procalcitonin (PCT) and Interleukine-6 (IL-6) were all elevated at the beginning of CRRT but were not associated with the formation of clogging (ΔI_WBC_: *p* = 0.725; ΔI_CRP_: *p* = 0.416; ΔI_PCT_: *p* = 0.414; ΔI_IL-6_: *p* = 0.358).

**Fig. 6:**
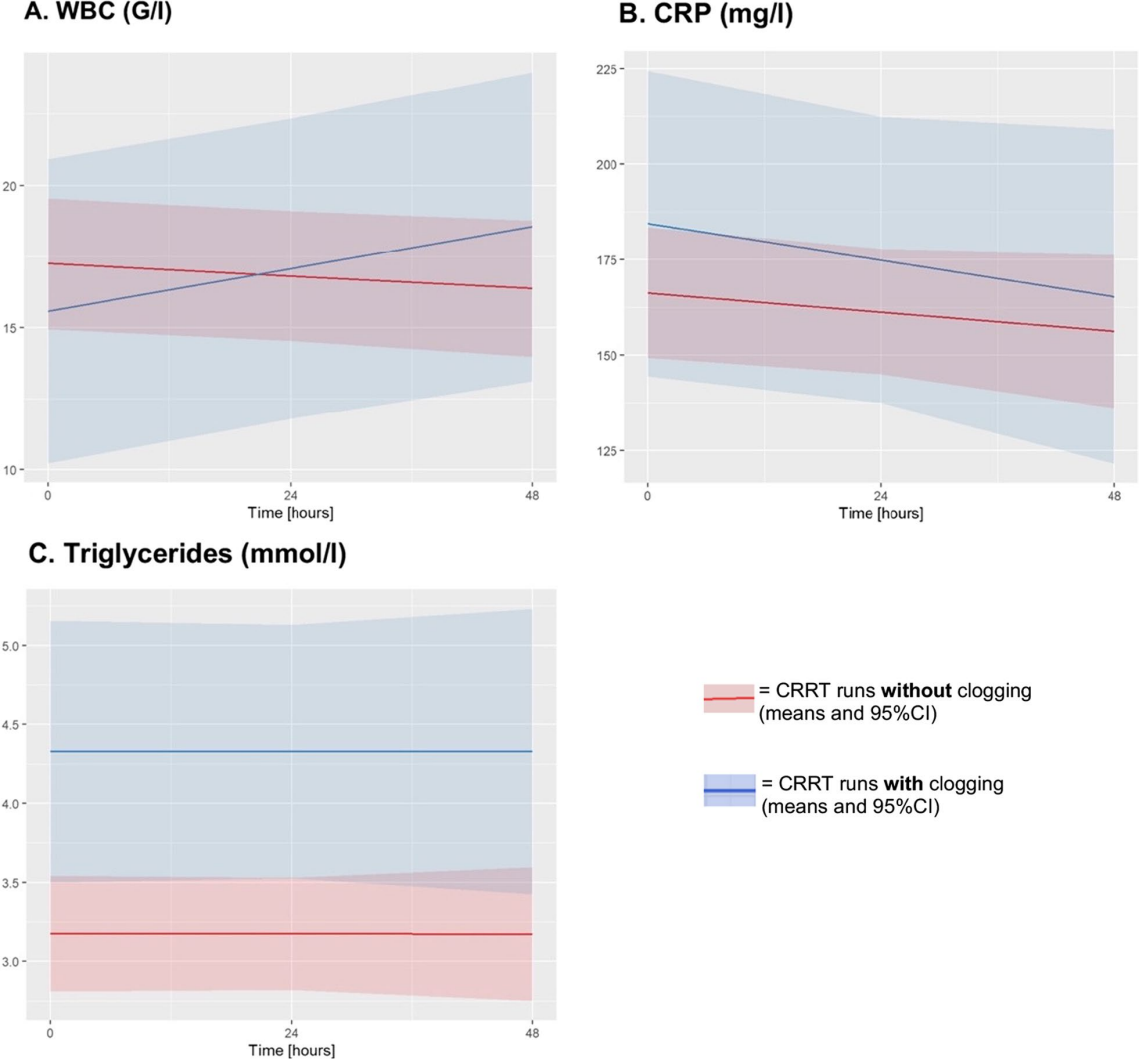
Factors associated with clogging formation—overall CRRT runs. Trajectories over time of factors associated with the formation of clogging (blue line) in comparison with filters without clogging (red line) in all assessed CRRT runs. The association of clogging with increasing WBC **A** without any concomitant increase in CRP levels **B** indicates an early inflammatory response phase. Triglyceride levels were remarkable elevated during CRRT runs with clogging **C**

WBCs showed a decreasing tendency over time during CRRT course with patent filters (non-clogging group), whereas an increase of leucocytes could be observed in association with clogging formation (Δβ: *p* = 0.002) (Fig. 6). Subgroup analysis revealed an additional distinct downwards-trending course of CRP over time in association with the occurrence of first clogging (Δβ: *p* = 0.045).

### Coagulation

There were no significant changes in thrombocyte counts, fibrinogen levels or anti-Xa activity over time in relationship with the formation of clogging (Δβ_Thrombocytes_: *p* = 0.414; Δβ_Fibrinogen_: *p* = 0.693; Δβ_Anti-Xa_: *p* = 0.826). Overall D-dimer levels increased during conduction of CRRT runs (β: *p* = 0.007) but the course was not associated with the occurrence of clogging (Δβ: *p* = 0.265).

### Triglyceride

Mixed regression model showed increased triglyceride plasma levels at first measurement after filter start in the clogging group (ΔI: *p* = 0.013) (Fig. 6). Daily amount of intravenous applied propofol and enteral nutrition were investigated as potential source of hypertriglyceridemia-induced clogging. Parenteral nutrition was not considered, since sample size was too low for reliable analysis (13 patients: 1 with clogging, 12 without clogging).

**Fig. 7:**
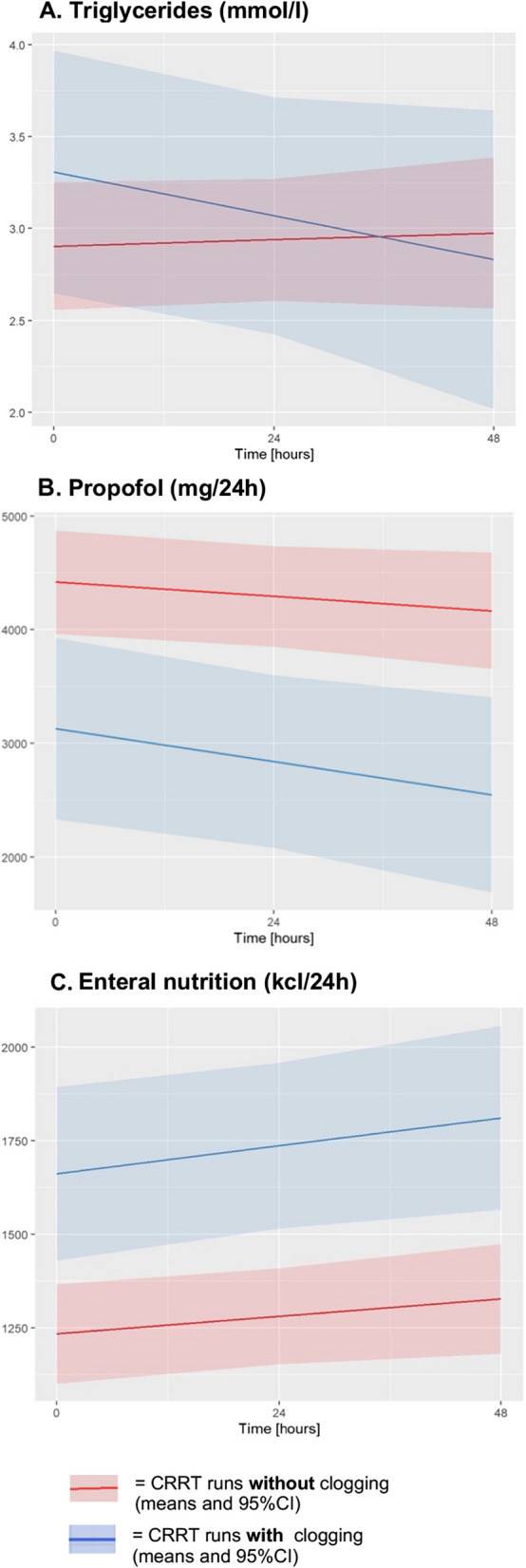
Factors associated with clogging formation in the first-clogging subgroup. While clogging was found to be associated with consistently elevated triglyceride plasma levels in the overall cohort, subgroup analysis of the first-clogging subgroup revealed an association with decreasing plasma levels over time **A**. In addition, first-clogging was attributed to reduced propofol exposure **B** and an increased enteral nutritional intake **C**. The quantity of enteral nutrition and propofol administered was measured as the cumulative dose received within the preceding 24 h, assessed at the initiation of the filter (T0), as well as at 24 h (T24) and 48 h (T48) thereafter

Average amount of daily received propofol or enteral nutrition (Promote® Fibres Plus, Abbott Laboratories, Chicago, IL, USA) did not differ substantially between filters with clogging compared to filters without in the overall cohort (ΔI_Propofol_: *p* = 0.277, Δβ_Propofol_ = 0.729; ΔI_Nutrition_: *p* = 0.153, Δβ_Nutrition_ = 0.384). Analyzing first-clogging in comparison with non-clogging CRRT runs manifested an association of clogging formation with reduced propofol exposure (ΔI: *p* = 0.006), but increased intake of enteral nutrition (ΔI: *p* = 0.002) in the 24 h before filter start (Fig. 7). No differences were observed for the total amount of applied propofol after ICU admission (*p* = 0.221).

## Discussion

The present study substantiated a notable incidence of metabolic alterations consistent with clogging among patients diagnosed with COVID-19 and successfully identified 4 factors associated with its occurrence: (1) type of CRRT device, (2) hypertriglyceridemia, (3) augmented enteral nutritional intake and (4) a time-dependent increment of WBC.

The occurrence of clogging, a phenomenon that has been under-represented in the critical care literature, has recently been investigated in two studies conducted during the pandemic years. Wen and colleagues were the first authors describing a reduction in lifespan cycles of SLEDs (2008 K/K2, Fresenius Medical Care) in patients with COVID-19 and association with a rise in the TMP, a parameter of reduced filter patency [11]. Comparable findings were reported by Khadzhynov et al. for CRRT devices (mFT, Fresenius Medical Care) with CVVHD modality using high-flux dialyzer filters (AV1000, Fresenius Medical Care) [13]. Both teams attributed an increased occurrence of clogging in critically ill patients with COVID-19 in comparison with a non-COVID control cohorts [11, 13]. Compared to these previous works, the hereby presented study focuses predominantly on the assessment of contributing factors to clogging pathophysiology to gain an improved understanding of the underlying mechanism.

A primary challenge in the assessment of clogging constitutes the lack of unified and objective criteria, bearing the risk of heterogeneity in the diagnosis within clinical practice.

The TMP, a calculated parameter provided by CRRT devices, serves as a conventional surrogate for assessing filter patency. However, the TMP is not solely determined by the permeability of the membrane but is also influenced by various factors, including blood flow, ultrafiltration, and the flow rate of the filtrate through the membrane. Consequently, it may not adequately reflect changes in membrane permeability.

As demonstrated in our findings, the TMP exhibits a strong dependence on the specific CRRT devices and associated modalities employed. In the context of CVVHDF, a pressure gradient is established between the blood and dialysate compartments to facilitate the filtration of fluid components from the blood through the membrane. In cases where filter settings remain constant, a continuous increase in TMP may signify a reduction in membrane patency.

Conversely, during isolated CVVHD, the TMP remains relatively low, because no pressure gradient is generated across the membrane. Consequently, a rise in TMP occurs only in a late stage of advance membrane obstruction.

Therefore, it becomes evident that new definitions are necessary to enable the early detection of impaired filter patency in CRRT devices, particularly those utilizing CVVHD modalities.

Khadzhynov et al. were the first to propose the use of metabolic changes as a marker of clogging in devices with RCA [13]. For our protocol we relied on an adapted version using metabolic alkalosis, hypernatremia and corrected_Alb_ Ca levels and excluding the CaSR. We chose to use corrected_Alb_ Ca levels instead of CaSR due to the rationale that the former provides a more accurate representation of true calcium levels. This preference stems from the fact that CaSR is typically regulated based on ionized calcium (iCa) levels in plasma and is hence influenced by albumin levels as well as the liver's capacity to metabolize citrate.

Previous authors also employed the normalization of metabolic derangements after a filter change as an indicator of clogging in the preceding filter [13]. However, we chose not to incorporate this criterion into our current study due to its limited generalizability, primarily resulting from the frequent recurrence of clogging in successive filters. Nevertheless, it is important to note that normalizing after a filter change with unchanged settings can be a strong indicator of prior clogging and warrants consideration in future research endeavors.

An index, for the accuracy of the chosen criteria is the reduced filter clearance of urea observed in the first-clogging subgroup, demonstrating an association of the metabolic sequelae of sodium citrate alongside relevant impairment of filter clearance capacity for urea. The absence of a comparable pattern for the clearance of creatinine, is not fully understood and requires further assessment for the explanation of this phenomenon.

The study revealed elevated sodium and bicarbonate levels from the initiation of CRRTs in the clogging group within both the overall cohort and the first-clogging subgroup. These findings raise the possibility of pre-existing factors contributing to the metabolic imbalances observed. Notably, these patterns were not detected in the first-filter subgroup, suggesting that previously performed CRRTs may have influenced these metabolic differences at the initiation of subsequent treatments, supporting the hypothesis that clogging might represent an accumulative entity spanning multiple consecutive CRRT runs.

The most notable factor associated with the phenomenon of clogging was the reported variance in its incidence between CRRT devices. Since the two devices used at our institution were run with different filters as well as different modalities (CVVHD for mFT and CVVHDF for PFS), the presented data are not conclusive, whereas filter membranes or modalities are predominantly responsible for observed divergences. However, recent clinical experience gathered after conduction of this study, manifested the occurrence of clogging even with CVVHDF on mFT devices. This raises the question of filter hemocompatibilities of the used dialysis membranes—an important aspect of any filter patency [14]. Contact of filter membranes with human blood always leads to the absorption of plasma proteins resulting in the formation of a secondary membrane. This plasma membrane consists commonly of proteins, such as albumin and fibrinogen, and is formed within minutes after primary contact, contributing to a rapid decrease of membrane permeability [15]. Hydrophilic synthetic filter membranes, such as polyulfone (PSF) and polyethersulfone (PES) membranes, were employed to enhance hemocompatibility and minimize secondary membrane formation [14, 16]. Notwithstanding this advancements made in synthetic dialyzer membranes, adverse interactions between blood and the membrane persist, and our knowledge regarding specific patient-related factors contributing to these interactions remains limited [16, 17].

Our cohort exhibited a notable elevation in triglyceride levels associated with the development of clogging, as a potential contributing factor. Kazory and colleagues reported in 2008 for the first time a case of a patient with tenacious circuit failures during CRRT, associated with hypertriglyceridemia [6]. Since then, 6 additional case reports have been published associating elevated plasma lipid levels with an impairment of filter function [7–9, 18–20]. With this study, we have demonstrated, for the first time on a larger cohort, a direct association between clogging and hypertriglyceridemia, providing evidence beyond isolated case reports. Contrary to our expectations and previous reports, our findings revealed an association between clogging and the daily cumulative amount of enteral nutrition (kcl/d), rather than with intravenous administration of lipid-enriched solutions, as potential cause of the hypertriglyceridemia [6, 7, 18]. The observed reduction in the rate of propofol (mg/d) administration during the onset of clogging might be attributed to reactive measures, likely in response to the increased lipid levels. These findings indicate the requirement for reassessment of nutritional practice in critical care.

A further finding was the elevated inflammatory parameters in patients on CRRT. The identified divergent pattern in leucocyte levels, marked by a decrease in non-clogging patients and an increase in the presence of clogging formation, along with a declining trend in IL-6, PCT, and CRP levels, provides evidence supporting an association between clogging and the occurrence of a recurrent inflammatory process. This is consistent with previous studies which have hypothesized a potential influence of heightened inflammatory burden on the occurrence of clogging [11]. Whereas the observed relapsing inflammation might contribute to a reduction of the hydrophilic characteristics of the AV1000 membranes, increasing susceptibility for the formation of a lipophilic secondary membrane in a triglyceride-rich environment, might be a possible explanation for the rapid decrease of hemocompatibility, but has to be further investigated for a conclusive evaluation.

Clinicians working with susceptible devices should be aware of the increased risk of clogging formation, especially in the context of hypertriglyceridemia and relapsing inflammatory response. Regular monitoring for indications of metabolic abnormalities and ensuring that triglyceride levels remain within the normal range could aid in the timely detection and prevention of potential complications. Change of circuits should be considered in case of refractory metabolic alterations attributable to clogging.

### Limitations

Our study is subject to several limitations that should be taken into account when interpreting the results. The retrospective design poses challenges in establishing causal relationships and controlling for confounding variables. In addition, the lack of standardized criteria for identifying clogging, coupled with the arbitrary criteria we utilized, introduces subjectivity and increases the potential for misclassification. This is specially a risk in patients with impaired liver function, where the metabolic conversion from citrate to bicarbonate is impaired. The increased citrate exposure in clogged filters may introduce a confounding factor, but it seems to be primarily mediated by the device type. This difference may be due to lower post-filter iCa targets in the mFT compared to the PFS protocol (Additional file 1: Tables S2 and S3 in the supplementary section). When focusing solely on mFT devices, the residual difference between clogged and non-clogged filters appears too small to have a realistic clinical impact and might be a response to potential higher post-filter iCa levels resulting from calcium accumulation rather than a cause of the metabolic derangements. Moreover, the analysis of isolated CRRT runs may result in patient-related confounders rather than CRRT-associated factors. Moreover, the existence of missing values may have an impact on the statistical power of specific analyses.

## Conclusion

This study confirms the high prevalence of metabolic derangements during CRRT indicative for clogging in a critically ill COVID-19 cohort and demonstrates a particular susceptibility of specific CRRT devices. The observed distinction among devices may be attributed to variations in the hemodialysis filters or the chosen modalities. Moreover, we found that clogging might be associated with increasing WBC and hypertriglyceridemia that could be explained by enteral nutrition. However, the precise underlying mechanism responsible for the occurrence of clogging remains elusive and warrants further investigations to enhance our understanding, prevent its onset, and develop novel strategies to enhance the efficacy of CRRTs in critically ill patients.

### Supplementary Information

**Additional file 1.** **Table S1. **Missing values. **Table S2.** Protocol CRRT - PFS.** Table S3.** Protocol CRRT - mFT. **Table S4.** Mixed effect model analyzing the effect of clogging on time-varying trajectories of sodium, bicarbonate and albumin-corrected calcium levels (correctedAlb Ca) as well as calcium substitution rate (CaSR.). Results present the impact of clogging on intercept (ΔI) and β-coefficient (Δβ) in comparison to non-clogging CRRT runs. *p<0.05, **p<0.01, ***p<0.001. **Table S5.** Estimates and 95%CI of the association between accumulative citrate exposure and clogging were calculated for the overall cohort (Model 1) and isolated mFT devices (Model 3). Model 2 displays the effect of PFS compared to mFT devices on citrate exposure. No significant differences in CRRT dosage were observed between filters with and without clogging. *p<0.05, **p<0.01, ***p<0.001. **Table S6.** Mixed effect model representing timed-dependent trajectories of renal biomarkers and factors with potential impact on clogging formation. Results present the impact of clogging on intercept (ΔI) and β-coefficient (Δβ) in comparison to non-clogging CRRT runs. Clogging was associated with increased plasma triglyceride levels. Analysis of first filters with clogging (first-clogging group, right columns) revealed a higher enteric nutritional intake in patients during the first onset of clogging. *p<0.05, **p<0.01, ***p<0.001. **Figure S1.** TMP.

## Data Availability

The data sets generated and analyzed during the current study are not publicly available, since they are currently in use for follow-up analysis but are available from the corresponding author on reasonable request.

## Abbreviations

AKI: Acute kidney injury
CaSR: Calcium substitution rate
Corrected_Alb_ Ca: Total albumin-corrected calcium
COVID-19: Coronavirus disease 2019
CRP: C-reactive protein
CRRT: Continuous renal replacement therapy
CVVHD: Continuous veno-venous hemodialysis
CVVHDF: Continuous veno-venous hemodiafiltration
iCa: Ionized calcium
ICU: Intensive care unit
IHD: Intermittent hemodialysis
IL-6: Interleukine-6
mFT: MultiFiltrate
PCR: Polymerase chain reaction
PCT: Procalcitonin
PES: Polyethersulfone
PFS: Prismaflex System
PSF: Polysulfone
PVP: Polyvinylpyrrolidone
RCA: Regional citrate anticoagulation
SARS-CoV-2: Severe acute respiratory syndrome coronavirus 2
SLED: Sustained low efficiency dialysis
SOFA score: Sequential organ failure assessment score
TMP: Transmembrane pressure
WBC: White blood cell counts
