# Supplementary material for: Metabolic disturbances potentially attributable to clogging during continuous renal replacement therapy
Source: Intensive Care Med Exp. 2023 Dec 21;11:99. doi: 10.1186/s40635-023-00581-9 (PMC10739685; doi:10.1186/s40635-023-00581-9)
Supplement: Supplementary file 1 — Additional file 1. Table S1. Missing values. Table S2. Protocol CRRT - PFS. Table S3. Protocol CRRT - mFT. Table S4. Mixed effect model analyzing the effect of clogging on time-varying trajectories of sodium, bicarbonate and albumin-corrected calcium levels (correctedAlb Ca) as well as calcium substitution rate (CaSR.). Results present the impact of clogging on intercept (ΔI) and β-coefficient (Δβ) in comparison to non-clogging CRRT runs. *p<0.05, **p<0.01, ***p<0.001. Table S5. Estimates and 95%CI of the association between accumulative citrate exposure and clogging were calculated for the overall cohort (Model 1) and isolated mFT devices (Model 3). Model 2 displays the effect of PFS compared to mFT devices on citrate exposure. No significant differences in CRRT dosage were observed between filters with and without clogging. *p<0.05, **p<0.01, ***p<0.001. Table S6. Mixed effect model representing timed-dependent trajectories of renal biomarkers and factors with potential impact on clogging formation. Results present the impact of clogging on intercept (ΔI) and β-coefficient (Δβ) in comparison to non-clogging CRRT runs. Clogging was associated with increased plasma triglyceride levels. Analysis of first filters with clogging (first-clogging group, right columns) revealed a higher enteric nutritional intake in patients during the first onset of clogging. *p<0.05, **p<0.01, ***p<0.001. Figure S1. TMP. [file 40635_2023_581_MOESM1_ESM.docx]

**Additional file 1**

**Table S1: Missing values**

| **Values measured every 24h** | |  |  |  |  |  |  |  |  |  |
| --- | --- | --- | --- | --- | --- | --- | --- | --- | --- | --- |
| **Total Datapoints** |  | Fibrinogen | D-dimer | Thrombocytes | anti-Xa | correctedAlb Ca | CRP | PCT | Leucocytes |  |
| **564** | Missing Absolute | 139 | 180 | 55 | 74 | 64 | 63 | 66 | 54 |  |
|  | Missing Percentage | 24.65 | 31.91 | 9.75 | 13.12 | 11.35 | 11.17 | 11.70 | 9.57 |  |
|  |  |  |  |  |  |  |  |  |  |  |
|  |  | IL-6 | Triglyceride | Propofol | Enteral nutrition | Creatinine | Urea | Phosphate | Magnesium |  |
|  | Missing Absolute | 389 | 391 | 0 | 0 | 2 | 6 | 0 | 0 |  |
|  | Missing Percentage | 68.97 | 69.33 | 0.00 | 0.00 | 0.35 | 1.06 | 0.00 | 0.00 |  |
|  |  |  |  |  |  |  |  |  |  |  |
|  |  |  |  |  |  |  |  |  |  |  |
| **Values measured every 8h** | |  |  |  |  |  |  |  |  |  |
| **Total Datapoints** |  | TMP | iCa | CaSR | Sodium | HCO3 | Potassium |  |  |  |
| **1581** | Missing Absolute | 2 | 0 | 31 | 5 | 6 | 5 |  |  |  |
|  | Missing Percentage | 0.00 | 0.00 | 0.02 | 0.00 | 0.00 | 0.00 |  |  |  |

**Table S2: Protocol CRRT - PFS**

| **Weight (kg)** | **50** | **60** | **70** | **80** | **90** | **100** |
| --- | --- | --- | --- | --- | --- | --- |
| Blood flow [mL/min] | 100 | 120 | 120 | 120 | 120 | 120 |
| Substituate post filter [mL/h] | 250 | 250 | 250 | 500 | 500 | 500 |
| Dialysate [mL/h] | 500 | 700 | 900 | 1000 | 1200 | 1300 |
| **PBP (mL/h)** |  |  |  |  |  |  |
| Citrate 2.5 mmol/L blood | 833 | 1000 | 1000 | 1000 | 1000 | 1000 |
| Citrate 3 mmol/L blood | 1000 | 1200 | 1200 | 1200 | 1200 | 1200 |
| Citrate 3.5 mmol/L blood | 1167 | 1400 | 1400 | 1400 | 1400 | 1400 |
| Ca++-Substitution in % of the filtrated amount of Ca++- | 100 | 100 | 100 | 100 | 100 | 100 |
| **CRRT dose (ml/kg KG/h)** |  |  |  |  |  |  |
| Citrate 2.5 mmol/L blood | 32 | 33 | 31 | 31 | 30 | 28 |
| Citrate 3 mmol/L blood | 35 | 36 | 34 | 34 | 32 | 30 |
| Citrat 3.5 mmol/L blood | 38 | 39 | 36 | 36 | 34 | 32 |
|  |  |  |  |  |  |  |
| **Targeted postfilter iCa** | 0.25 – 0.5 mmol/L | |  |  |  |  |

**Table S3: Protocol CRRT - mFT**

| **Targeted CRRT dose** |  | **Body weight** | (kg) |  |  |  |  |  |  |  |
| --- | --- | --- | --- | --- | --- | --- | --- | --- | --- | --- |
|  |  | 50 | 60 | 70 | 80 | 90 | 100 | 110 | 120 | 130 |
| **20 ml/kg/h** | **Dialysate (ml/h)** | 1000 | 1200 | 1400 | 1600 | 1800 | 2000 | 2100 | 2200 | 2300 |
|  | **Blood flow (ml/min)** | 50 | 60 | 70 | 80 | 90 | 100 | 100 | 110 | 110 |
| **25 ml/kg/h** | **Dialysat (ml)** | 1250 | 1500 | 1750 | 2000 | 2250 | 2500 | 2600 | 2700 | 2800 |
|  | **Blood flow (ml/min)** | 70 | 80 | 90 | 100 | 110 | 120 | 130 | 140 | 150 |
| **30 ml/kg/h** | **Dialysat (ml)** | 1500 | 1800 | 2100 | 2400 | 2700 | 3000 | 3100 | 3200 | 3300 |
|  | **Blood flow (ml/min)** | 80 | 90 | 110 | 120 | 130 | 150 | 160 | 160 | 170 |
|  |  |  |  |  |  |  |  |  |  |  |
| **Targeted postfilter iCa** | 0.25 – 0.34 mmol/L |  |  |  |  |  |  |  |  |  |

**Table S4:**

| **Predictors** | | **Regression model** | | | | **Effect of clogging** | | | |  |
| --- | --- | --- | --- | --- | --- | --- | --- | --- | --- | --- |
|  |  | Intercept | Time (h)  1st degree | Time (h)  2nd degree | Time (h)  3rd degree | Intercept (ΔI) | Time (h)  1st degree (Δβ) | Time (h)  2nd degree (Δβ^2^) | Time (h) 3rd degree (Δβ^3^) |  |
|  |  |  |  |  |  |  |  |  |  |  |
|  |  |  |  |  |  |  |  |  |  |  |
| **Markers clogging:** | |  |  |  |  |  |  |  | |  |
|  | Sodium (mmol/l) | **140.47***** | 0.36 | **-2.03*** | -0.03 | **5.29***** | 2.14 | 3 | -0.92 |  |
|  |  | [139.74 – 141.20] | [-0.86 – 1.58] | [-3.81 – -0.26] | [-1.40 – 1.35] | [3.42 – 7.16] | [-0.54 – 4.82] | [-1.00 – 7.00] | [-3.52 – 1.68] |  |
|  | Bicarbonate (mmol/l) | **23***** | 0.15 | 2.37 | 0.51 | 1.62 | 3.18 | 4.14 | -0.25 |  |
|  |  | [22.34 – 23.91] | [-1.46 – 1.76] | [0.04 – 4.70] | [-1.31 – 2.32] | [-0.38 – 3.62] | [-0.41 – 6.78] | [-1.21 – 9.48] | [-3.70 – 3.19] |  |
|  | corrected_Alb_ Ca (mmol/l) | **2.75***** | 0 |  |  | 2.57 | 0 |  |  |  |
|  |  | [2.43 – 3.07] | [0.00 – 0.00] |  |  | [2.48-2.66] | [-0.00 – 0.00] |  |  |  |
|  |  |  |  |  |  |  |  |  |  |  |
|  | CaSR. | **1.78***** | **0.4*** | 0.07 | -0.37 | -0.05 | -0.41 | 0.21 | 0.32 |  |
|  | (mmol/l) | [2.60 – 1.96] | [0.05 – 0.75] | [-0.43 – 0.58] | [-0.76 – 0.01] | [-0.39 – 0.01] | [-1.02 – 0.19] | [-0.68 – 1.10] | [-0.27 – 0.91] |  |
|  |  |  |  |  |  |  |  |  |  |  |
| **Subgroup first clogging:** | |  |  |  |  |  |  |  | |  |
|  | Sodium (mmol/l) | **139.63***** | 0.83 | **-2.83*** | 0.08 | **4.77***** | -0.22 | 3.33 | -1.19 |  |
|  |  | [138.68 – 140.57] | [-0.84 – 2.51] | [-5.22 – -0.43] | [-1.75 – 1.9] | [2.89 – 6.65] | [-3.27 – 2.82] | [-1.14 – 7.80] | [-4.34 – 1.96] |  |
|  | Bicarbonate (mmol/l) | **23.16***** | -0.06 | 1.84 | 0.06 | 0.97 | 2.15 | 4.12 | 0.22 |  |
|  |  | [22.09 – 24.24] | [-2.30 – 2.18] | [-1.33 – 5.02] | [-2.39 – 2.50] | [-1.17 – 3.11] | [-1.97 – 6.27] | [-1.88 – 10.12] | [-3.99 – 4.44] |  |
|  | corrected_Alb_ Ca (mmol/l) | **2.86***** | 0 |  |  | -0.29 | 0 |  |  |  |
|  |  | [2.42 – 3.29] | [-0.00 – 0.00] |  |  | [-1.16 – 0.58] | [-0.00 – 0.00] |  |  |  |
|  |  |  |  |  |  |  |  |  |  |  |
|  | CaSR. | **1.81***** | **0.36***** | 0 |  | -0.11 | **-0.31**** | **-0.17*** |  |  |
|  | (mmol/l) | [1.70 – 1.93] | [0.22 – 0.50] | [-0.11 – 0.11] |  | [-0.28 – 0.06] | [-0.51 – -0.12] | [-0.32 – -0.01] |  |  |
|  |  |  |  |  |  |  |  |  |  |  |
|  |  |  |  |  |  |  |  |  |  |  |

Mixed effect model analyzing the effect of clogging on time-varying trajectories of sodium, bicarbonate and albumin-corrected calcium levels (corrected_Alb_ Ca) as well as calcium substitution rate (CaSR.). Results present the impact of clogging on intercept (ΔI) and β-coefficient (Δβ) in comparison to non-clogging CRRT runs. *p<0.05, **p<0.01, ***p<0.001

**Table S5.**

|  |  | **Citrate target and CRRT dose** | | | |  |  |  |
| --- | --- | --- | --- | --- | --- | --- | --- | --- |
| **Predictors** | | **Regression model** | | **Effect of clogging** | | **Effect of PFS devices** | |  |
|  |  | Intercept (I) | Time (h)  1st degree  (β) | Intercept (ΔI) | Time (h)  1st degree (Δβ) | Intercept (ΔI) |  |  |
|  |  |  |  |  |  |  | Time (h)  1st degree (Δβ) |  |
|  |  |  |  |  |  |  |  |  |
| **Citrate traget (mmol)** | |  |  |  |  |  |  |  |
|  | Model 1 (effect of clogging) | **202.62***** | **27.48***** | 1.11 | **4.72***** |  |  |  |
|  |  | [175.63 – 229.61] | [27.09 – 27.86] | [-68.41 – 70.62] | [3.84 – 5.60] |  |  |  |
|  | Model 2 (effect of PFS devices) | **220.18***** | **31.34***** |  |  | -36.69 | **-7.32***** |  |
|  |  | [192.41 – 247.94] | [30.95 – 31.74] |  |  | [-78.45 – 5.07] | [-7.94 – -6.69] |  |
|  | Model 3 (effect of clogging,only in mFT devices) | **227.35*****  [191.11 – 263.60] | **30.92*****  [30.47 – 31.37] | -23.21  [-93.02 – 46.60] | **1.28***  [0.49 – 2.07] |  |  |  |
| **CRRT dose (ml/kg/h)** | |  |  |  |  |  |  |  |
|  |  | **24.49***** | **-0.21***** | 2.28 | 0.06 |  |  |  |
|  |  | [23.02 – 25.96] | [ -0.25 – -0.18] | [-1.51 – 6.07] | [ -0.03 – 0.15] |  |  |  |

Estimates and 95%CI of the association between accumulative citrate exposure and clogging were calculated for the overall cohort (Model 1) and isolated mFT devices (Model 3). Model 2 displays the effect of PFS compared to mFT devices on citrate exposure. No significant differences in CRRT dosage were observed between filters with and without clogging. *p<0.05, **p<0.01, ***p<0.001

**Table S6.**

|  |  | **Renal biomarkers and factors potentially associated with clogging  formation** | | | | **Subgroup first-clogging** | |  |  |  |
| --- | --- | --- | --- | --- | --- | --- | --- | --- | --- | --- |
| **Predictors** | | **Regression model** | | **Effect of clogging** | | **Regression model** | | **Effect of clogging** | |  |
|  |  | Intercept (I) | Time (h)  1st degree  (β) | Intercept (ΔI) | Time (h)  1st degree (Δβ) | Intercept (I) | Time (h)  1st degree  (β) | Intercept (ΔI) | Time (h)  1st degree (Δβ) |  |
|  |  |  |  |  |  |  |  |  |  |  |
|  |  |  |  |  |  |  |  |  |  |  |
| **Renal biomarkers:** | |  |  |  |  |  |  |  |  |  |
|  | Creatinine (mmol/l) | **126.34***** | **-0.49***** | -4.18 | 0.19 | **125.3***** | -**0.54***** | 5.17 | 0.09 |  |
|  |  | [117.44 – 135.25] | [-0.64 – -0.35] | [-27.12 – 18.75] | [-0.14 – 0.51] | [114.57 – 136.02] | [-0.72 – -0.37] | [-15.93 – 26.27] | -0.24 – 0.42] |  |
|  | Urea (mmol/l) | **12.5***** | **-0.05***** | 1.5 | 0.03 | **11.6***** | **-0.07***** | **2.71**** | **0.04*** |  |
|  |  | [11.18 – 12.91] | [-0.07 – -0.04] | [-0.74 – 3.73] | [-0.00 – 0.06] | [10.60 – 12.61] | [-0.08 – -0.05] | [0.73 – 4.69] | [0.01 – 0.08] |  |
|  |  |  |  |  |  |  |  |  |  |  |
| **Inflammation:** | |  |  |  |  |  |  |  |  |  |
|  | WBC (G/l) | **17.25***** | -0.02 | -1.67 | **0.08**** | **18.38***** | **-0.03*** | -1.09 | 0.07 |  |
|  |  | [14.95 – 19.55] | [-0.04 – 0.00] | [-7.48 – 4.14] | [0.03 – 0.13] | [15.33 – 21.44] | [-0.06 – -0.00] | [-7.15 – 4.98] | [0.01 – 0.12] |  |
|  | CRP (mg/l) | **166.34***** | -0.21 | 18.05 | -0.19 | **151.01***** | -0.06 | 30.88 | **-0.96*** |  |
|  |  | [149.23 – 183.44] | [-0.59 – 0.16] | [-25.50 – 61.60] | [-1.05 – 0.68] | [130.05 – 171.96] | [-0.55 – 0.44] | [-10.88 – 72.65] | [-1.90 – -0.02] |  |
|  | PCT (ng/ml) | **10.54***** | -0.06 | 6.05 | -0.04 | **8.15**** | -0.02 | 2.62 | -0.04 |  |
|  |  | [4.90 – 16.18] | [-0.13 – 0.01] | [-8.49 – 20.59] | [-0.20 – 0.13] | [2.55 – 13.75] | [-0.08 – 0.04] | [-8.64 – 13.88] | [-0.16 – 0.07] |  |
|  | IL-6 (pg/ml) | **607.51**** | -3.77 | -416.01 | 3.64 | **663.78**** | -4.39 | -522.3 | 4.68 |  |
|  |  | [185.71 – 1029.32] | [-17.23 – 9.69] | [1833.71 – 1001.70] | [32.38 – 39.66] | [213.86 – 1113.70] | [-18.52 – 9.75] | [-1641.08 – 596.49] | [-27.11 – 36.46] |  |
|  |  |  |  |  |  |  |  |  |  |  |
| **Coagulation** | |  |  |  |  |  |  |  |  |  |
|  | Thrombocytes (G/l) | **200.52***** | -0.16 | -15.25 | -0.05 | **204.14***** | -0.19 | -15.78 | 0.12 |  |
|  |  | [183.80 – 217.23] | [-0.35 – 0.04] | [183.80 – 217.23] | [-0.49 – 0.39] | [185.05 – 223.22] | [-0.43 – 0.06] | [-53.71 – 22.14] | [-0.33 – 0.58] |  |
|  | Fibrinogen (g/l) | **6.30***** | 0.00 | 0.38 | -0.01 | **6.36***** | 0 | -0.36 | 0.00 |  |
|  |  | [5.96 – 6.64] | [-0.01 – 0.00] | [-0.49 – 1.24] | [-0.02 – 0.0] | [5.97 – 6.75] | [-0.01 – 0.01] | [-1.14 – 0.41] | [-0.01 – 0.01] |  |
|  | D-dimer (mg/l) | **7.05***** | **0.02**** | -0.78 | -0.01 | **6.96***** | **0.02**** | 0.73 | -0.02 |  |
|  |  | [6.15 – 7.96] | [0.01 – 0.03] | [-3.34 – 1.77] | [-0.05 – 0.02] | [5.83 – 8.09] | [0.01 – 0.04] | [-1.74 – 3.21] | [-0.05 – 0.02] |  |
|  | Anti-Xa activity (E/ml) | **0.38***** | 0.00 | -0.05 | 0.00 | **0.39***** | 0.00 | -0.04 | 0.00 |  |
|  |  | [0.35 – 0.41] | [-0.00 – 0.00] | [-0.12 – 0.02] | [-0.00 – 0.00] | [0.36 – 0.43] | [-0.00 – 0.00] | [-0.11 – 0.02] | [-0.00 – 0.00] |  |
|  |  |  |  |  |  |  |  |  |  |  |
| **Lipids** | |  |  |  |  |  |  |  |  |  |
|  | Triglycerides (mmol/l) | **3.18***** | 0.00 | **1.15*** | 0.00 | **2.9***** | 0.00 | 0.4 | -0.01 |  |
|  |  | [2.81 – 3.54] | [-0.01 – 0.01] | [-0.24 – 2.06] | [-0.02 – 0.02] | [2.56 – 3.25] | [-0.01 – 0.01] | [-0.34 – 1.15] | [-0.03 – 0.01] |  |
|  | Propofol  (mg in 24h) | **4035.41***** | -3.73 | -572.63 | -2.72 | **4418.15***** | -5.24 | **-1288.02**** | -6.92 |  |
|  |  | [3633.93 – 4436.90] | [-10.25 – 2.79] | [-1606.73 – 461.47] | [-18.15 – 12.71] | [3961.66 – 4874.65] | [-13.67 – 3.20] | [-2207.68 – -368.36] | [-23.06 – 9.21] |  |
|  | Enteral nutrition (kcal in 24h) | **1301.00***** | **2.00**** | 216.90 | -1.98 | **1233.36***** | 1.96 | **428.72**** | 1.15 |  |
|  |  | [1185.62 – 1416.38] | [0.11 – 3.89] | [-80.77 – 514.57] | [-80.77 – 514.57] | [1100.98 – 1365.75] | [-0.31 – 4.23] | [161.52 – 695.92] | [-3.19 – 5.49] |  |
|  |  |  |  |  |  |  |  |  |  |  |

Mixed effect model representing timed-dependent trajectories of renal biomarkers and factors with potential impact on clogging formation. Results present the impact of clogging on intercept (ΔI) and β-coefficient (Δβ) in comparison to non-clogging CRRT runs. Clogging was associated with increased plasma triglyceride levels. Analysis of first filters with clogging (first-clogging group, right columns) revealed a higher enteric nutritional intake in patients during the first onset of clogging. *p<0.05, **p<0.01, ***p<0.001

**Figure S1: TMP**


Clogging was initially linked to elevated TMP values, but it ultimately led to a slower rate of increase compared to non-clogging filters (A). In contrast, TMP levels in PFS devices showed an upward trend over time, while TMP levels in CVVHD modalities remained relatively lower (B).
